# Health service use in indigenous Sami and non-indigenous youth in North Norway: A population based survey

**DOI:** 10.1186/1471-2458-9-378

**Published:** 2009-10-08

**Authors:** Anne Lene Turi, Margrethe Bals, Ingunn B Skre, Siv Kvernmo

**Affiliations:** 1 Department of Psychology, University of Tromsø, N-9037 Tromsø, Norway; 2 Department of Child and Adolescent Psychiatry, University Hospital of North Norway, 9038 Tromsø, Norway; 3 Center of Child and Adolescent Mental Health-Region North, Department of Community Medicine, Faculty of Medicine, University of Tromsø, N- 9037 Tromsø, Norway

## Abstract

**Background:**

This is the first population based study exploring health service use and ethno-cultural factors in indigenous Sami and non-Sami youth in North Norway. The first aim of the present study was to compare the frequency of health service use between Sami adolescents and their non-indigenous peers. The second aim was to explore the relationships between health service use and ethno-cultural factors, such as ethnic context, Sami self-identification, perceived discrimination and Sami language competence. Finally, we wanted to explore the relationship between use of health services and emotional and behavioural problems.

**Method:**

The Norwegian Arctic Adolescent Health Study was conducted among 10th graders (15-16 years old) in junior high schools in North Norway. The sample consisted of 4,449 adolescents, of whom 450 (10.1%) were indigenous Sami and 3,999 (89.9%) were non-Sami.

**Results:**

Sami and non-Sami youth used all health services with equal frequency. However, several ethno-cultural factors were found to influence health service use. Sami youth in more assimilated ethnic contexts used general practitioners more than non-Sami youth. Youth with Sami self-identification had a higher probability of using the school health service compared with other youth. Ethnic barriers to health service use were also identified. Sami speaking youth with a high degree of perceived discrimination had lower probability of using school health services than non-Sami speaking youth. Sami youth with conduct problems were less likely than non-Sami to use psychologist/psychiatrist. The present study demonstrated a relationship between health need and actual health service use.

**Conclusion:**

Culture-specific factors influenced the help-seeking process in indigenous youth; some factors acted as barriers against health service use and other factors increased the probability of health service use.

## Background

Through history, the indigenous peoples of the Arctic have been exposed to forced assimilation, discrimination and prejudice from the dominant society, of which the health service is a part. Since there is limited availability of culture-specific health services to the indigenous population, negative attitudes towards the use of health services and a different help-seeking behaviour may be present in indigenous peoples, such as the Sami, compared with the majority population. To our knowledge, the present study is the first to focus on indigenous Sami adolescents and health service use.

### The indigenous Sami

The Sami are the indigenous people residing in the circumpolar parts of Norway, Sweden, Finland and the Russian Kola Peninsula, and are estimated to comprise around 70-100,000 individuals in these four different countries. Approximately 40,000 Sami people live in the Norwegian part of "Sapmi", the Sami homeland [1]. In North Norway the population is generally stable, the socio-economic differences between ethnic groups are small, almost all settlements are rural or semi-rural, and the Sami minority represents a group with a specific history, culture and language [2].

After the mid 1800s, the official Norwegian assimilation policy, called the Norwegianization policy, was stepped up, and continued for at least a century. Sami were to be assimilated into Norwegian society and culture by the prohibition of Sami language use in schools and public areas, the implementation of boarding schools, the assigning of Norwegian names to properties which were to be bought, etc. [3,4]. This harsh assimilation and colonization has for many Sami, as for other indigenous peoples worldwide, led to an extensive loss of ethnic identity, ethnic language and traditional knowledge. After the Second World War, the official assimilation policy was replaced by an integration policy and gradually political, cultural and language revitalization processes began, including increased cultural awareness, use of Sami language and customs and the establishment of a Sami Parliament and several different Sami institutions.

The outcome of the Norwegianization policy and the ethnic revitalization movement had a different impact in different Sami regions or ethnic contexts. Today, some Sami regions have strong cultural and ethnic support and a high density of Sami [5]. In the high density ethnic contexts, Sami and Norwegian languages have equal status as official languages, and several Sami institutions reside here. In other regions, Sami are in the minority. In low and medium ethnic density contexts, there is less structural and practical support for Sami culture. Prejudice and ethnic conflicts, for instance about land rights and teaching in the Sami language, are still present. In spite of this, many Sami from low and medium density contexts have either regained or never lost their cultural awareness and ethnic pride. Simultaneously, having an insecure ethnic identity is relatively common [6,7]. Thus, Sami youth have different experiences of ethnic identity loss, loss of indigenous language, and they have different experiences of ethnic discrimination [8]. Consequently, it is not easy to make predictions about Sami adolescents' use of health services. The health service use will most likely vary with regard to the qualities and degrees of different correlates of ethnicity.

### Ethnicity, ethno-cultural factors and health service use

There is a discrepancy between the need for health services and the use of these services both by minority youth in general [9-11] and by indigenous youth [11,12]. Several studies have found that children and youth from indigenous peoples tend to be reluctant to seek help and have a low rate of use of health services [13,14].

Cauce and colleagues [10] have reviewed the existing literature on cultural and contextual influence on the mental help-seeking process of minority adolescents. They proposed a model with three stages of help-seeking behaviour: Problem Recognition, Decision to Seek Help and Service Selection. According to their model, cultural norms, values and beliefs interact with each stage of the help-seeking process. Cultures may vary regarding illness perception and tolerance or acceptance of different symptoms. Thus, problem recognition is influenced by cultural beliefs and norms. Other cultural barriers may arise at other stages of the help-seeking process. In research on indigenous people's barriers towards health service use, trust issues and negative attitudes towards the different health services are often emphasized [9,15]. The public health services are sometimes perceived as representatives of "White man" or the majority culture that through history has oppressed and persecuted their people, resulting in a deeply felt mistrust of health care institutions [16]. Facing the health services of the majority, the minority patient often has concerns about language barriers, cross-cultural understanding and about being discriminated against [11,12]. Finally, structural barriers, such as limited availability of health services, long distances and cost, are relevant for some minority patients [10-12].

Research on the indigenous Sami and health services has mostly focused on the adult population and on satisfaction with health services rather than actual use. Nystad, Melhus and Lund found that Sami speakers are less satisfied with municipal general practitioner services compared with Norwegian speakers [17]. Psychiatric hospital treatment and treatment satisfaction of Sami and non-Sami patients were compared, finding that Sami patients showed less satisfaction with many aspects of treatment, including treatment alliance, information and contact with staff [18]. Møllersen, Sexton and Holte investigated differences in mental health and use of mental health services among Sami and non-Sami patients in outpatient clinics [19]. They did not find poorer mental health, differences in drop-out rates, or differences in therapeutic alliances among the Sami, compared with the majority population.

### Emotional/behavioural problems and health service use

A wide range of factors is involved in the decision to seek help, including appraisal of a problem as something to seek help for, willingness to seek help, social norms that encourage such behaviour and availability of appropriate services [20,21]. Generally, young people are more likely to seek help when they recognize that they have emotional or behavioural problems and when they have the knowledge and skills to seek help [22]. Some studies report that adolescents with higher levels of psychological distress are more likely to seek professional help, compared to those with lower levels [21,23]. At the same time, epidemiological studies have shown that a substantial proportion of adolescents in the general population with significant levels of psychopathology do not receive specialist treatment for their problems [23,24]. In the present study, the correspondence between perceived mental health need measured by self-report and actual use of the different health services is investigated. An understanding of the relationship between need and service use may help find ways to reach the adolescents with high levels of problems who need professional help, but do not receive it [25].

### Health services in Norway

In Norway, the school health service and the general practitioner/physician are part of the primary health care system; the services are used for both mental and physical health problems [26]. The primary health services are located in the municipalities and are relatively easy to access. The general practitioners are accessible during the day, and patients have to pay a small fee for seeing them. The primary health care system provides each citizen with one particular and permanent general practitioner.

The school health service provides vaccinations and physical screening examinations. It is also possible for young people to get information on health issues, contraceptives and counselling when needed. All schools usually provide this service free of charge to their students. Adolescents are most likely to visit the school health service on their own initiative, while seeking help from the general practitioner probably more often happens through the assistance of parents.

Services from psychologists or psychiatrists are part of the specialist health service for mental health [26]. Patients are usually referred to a psychologist or a psychiatrist by a general practitioner. For children and adolescents, the service is free of charge. When the youth are referred to the specialist mental health service, they have to expect both waiting lists and perhaps long distance travel before receiving help.

The research questions for the present study can be stated in the following way:

1. Are there differences in frequencies of health service use between North Norwegian indigenous Sami adolescents and their non-indigenous peers?

2. What is the relationship between health service use and ethno-cultural factors, such as Sami self-identification, perceived discrimination and Sami language competence?

3. What is the relationship between adolescents' use of health services and emotional and behavioural problems? Is there a relationship between need for help and use of help?

## Methods

### Sample and procedure

From January 2003 to January 2005 the cross-sectional study "The Norwegian Arctic Adolescent Health Study" (NAAHS) was conducted among 10^th ^graders (15-16 years old) in junior high schools (292 schools in total) in the three northernmost counties in Norway: Finnmark (Response Rate: 71%), Troms (Response Rate: 82%) and Nordland (Response Rate: 88%). The total, 5,877 students were invited to participate and 4,880 accepted. The data collection was conducted and funded by the Center for Sami Health Research at the University of Tromsø and the Norwegian Institute of Public Health in collaboration.

The questionnaires were administered in classroom settings, monitored by project staff. Students who were not present at school completed the questionnaire at a later session. The students and their parents were given written information about the study; the students gave written consent. The study obtained approval and consent from the Regional Medical Ethical Committee, the Norwegian Data Inspectorate and the school authorities. Adolescents' ethnicity was categorized according to their reports on ethnic background variables. The non-responders (431 persons) to these questions were excluded from the analyses. Thus, the sample to be analysed in this study consisted of 4,449 adolescents, of whom 450 (10.1%) were indigenous Sami and 3,999 (89.9%) were non-Sami. The excluded individuals did not differ from the participants in reported frequency of health service utilization, nor were there differences regarding gender distribution and socio-economic status.

### Measures

#### Use of health services

The participants were given a list of health services and asked to rate the frequency of their visits: "Never", "1-3 times in the past 12 months", or "More than 4 times in the past 12 months". The variables were recoded into dichotomous variables with "Have used one or more times" and "Never used" for each of the health service options. The list of health services included the school health service, general practitioner/physician and psychologist/psychiatrist.

#### Socio-economic status (SES)

Participants were asked about both their mother's and father's occupations. This information was classified according to the International Standard Classification of Occupation ISCO-88 [27], which is also the basis for the Norwegian occupational standard. The nine categories were reclassified into five categories, based on the parent with the highest classified occupation: 1) Senior officials/managers, 2) Professionals/technicians, 3) Clerical workers, 4) Agriculture, fishery and reindeer herding and 5) Manual workers. The variable was dummy coded into high SES (the two highest rated occupations) and low SES (the other occupations).

#### Non-Sami and Sami ethnicity

Ethnicity was categorized using the method described by Kvernmo and Heyerdahl [5,28,29] and Aubert [2]. Participants reported parents' ethnicity separately for mothers, fathers and themselves by selecting from a list of ethnic groups, including five choices: "Sami", "Norwegian", "Finnish", "Kven" (Finnish-speaking immigrants from northern Finland and Sweden who settled in North Norway in the 1700s and 1800s), and "Other". The participants were allowed to select more than one option from the list. The participants were also given a list of languages and reported the language competence of grandparents, parents and of themselves. If grandparents' or parents' language was reported as Sami, or if parents' or their own ethnicity was reported as Sami, the ethnicity of the respondents was classified as Sami. Participants were classified as Sami if they had selected multiple ethnic categories, "Sami" being one. The non-Sami group was composed of all participants who reported a non-Sami affiliation, identity and language. Not all individuals who were classified as having Sami ethnicity reported subjective Sami self-identification.

*Sami self-identification *was classified according to the statement "I perceive myself as Sami", measured on a four-point scale from: (1) "I strongly agree", (2) "I agree", (3) "I disagree" to (4) "I strongly disagree". Respondents who strongly agreed or agreed perceiving themselves as Sami were categorized as having Sami self-identification.

#### Sami language competence

The adolescents were asked to report whether they had learned Sami language at home or not.

#### Ethnic context

The Sami context categories are based on density of Sami population, different levels of forced assimilation, loss of ethnic identity and language, stigmatization and revival of ethno-cultural values [30]. The high density context is located in the highland of Finnmark county. The medium density context is located on the coast of Finnmark, Troms and Nordland, and the low density context is located in the highland of Troms and Nordland counties.

#### Urban/rural area

Urban areas are the cities in North Norway and rural areas are the small towns and villages.

#### Strengths and Difficulties Questionnaire

Emotional and behavioural problems were measured by the "Strengths and Difficulties Questionnaire" (SDQ), a 25-item questionnaire with four problem scales and a pro-social subscale summing up to a total [31]. In our analyses we used the four problem scales of five items each: The hyperactivity subscale (Crohnbach's α for non-Sami = 0.63 and Sami = 0.66), the emotional symptoms subscale (Crohnbach's α for non-Sami = 0.70 and Sami = 0.64), the conduct problems subscale (Crohnbach's α for non-Sami = 0.47 and Sami = 0.51) and the peer problems subscale (Crohnbach's α for non-Sami = 0.52 and Sami = 0.52). Each one of the items can be rated "not true", "somewhat true" or "certainly true".

#### Alcohol use

The question "How often have you drunk alcohol during the last year?" had several options: "I have never used alcohol", "not at all during the last year", "a couple of times last year", "approximately once per month", "2-3 times per month", "once a week", "2-3 times a week" and "4-7 times a week". We recoded these variables into the following categories: "Rarely or never drinks", "1-3 times per month" and "1-7 times a week".

#### Perceived discrimination

The perceived discrimination scale consisted originally of nine items [32]. In this study only five items were used, assessing experiences of being teased or threatened, or feeling unaccepted because of one's ethnicity. The adolescents responded on a scale ranging from (1) strongly disagree to (4) strongly agree. The following statements were used: a) "I think others have behaved unfairly/negatively towards people from my culture", b) "I don't feel accepted by people from other cultures", c) "I feel that people from other cultures are against me", d) "I have been teased or insulted because of my ethnic background" and e) "I have been threatened and assaulted/attacked because of my ethnic background". The adolescents responded on a scale ranging from (1) strongly disagree to (4) strongly agree. The internal consistency on the five items was α = 0.81 for non-Sami youth and α = 0.83 for Sami youth, respectively.

### Statistical analysis

Chi-square tests were used for comparisons of categorical variables between groups. For ethical reasons, when N was lower than 10 in a cell, the ethnic differences were not reported. In such instances we replaced the data for each ethnic group with the total sum of both groups. The frequencies of health service use were compared between rural and urban areas in both ethnic groups, in order to rule out the possibility that ethnic differences in utilization arose from differences in access to health services in rural and urban areas.

Stepwise hierarchical logistic regression models were applied for the analysis of predictors for the use of the school health service, general practitioner and psychologist/psychiatrist. In the first step, demographic and ethno-cultural variables were included. In the second step, the main effects of emotional and behavioural problems were explored. Finally, as we wanted to explore the combined effect of the predictors, interaction terms were made. We wanted to explore whether the following were associated with the use of school health service and general practitioner: Sami self-identification combined with Sami language competence (Sami Self-Identification × Sami Language Competence) and perceived discrimination (Sami Self-Identification × Perceived Discrimination), and Sami language competence combined with the level of perceived discrimination (Sami Language Competence × Perceived Discrimination). The frequency of use of the psychologist/psychiatrist facility is low, and due to the lack of statistical power these three interactions were not included in the regression model for the use of psychologist/psychiatrist. Further, we wanted to explore whether ethnicity (Sami or non-Sami) combined with the level of emotional problems (Ethnicity × Emotional Problems), conduct problems (Ethnicity × Conduct Problems), peer problems (Ethnicity × Peer Problems), hyperactivity problems (Ethnicity × Hyperactivity) and use of alcohol (Ethnicity × Alcohol Use) were associated with the use of the school health service, general practitioner and psychologist/psychiatrist.

## Results

### Sample Characteristics

The gender distribution was equal between the Sami and non-Sami group (χ^2 ^(1) = 0.206, p = .650); among the Sami youth 50.7% (N = 228) were boys, and 49.5% (N = 1981) were boys in the non-Sami sample. The non-Sami subsample consisted of youth with both parents having only Norwegian ethnicity (90.6%), multi-ethnic Norwegian youth (Norwegian, Kven/Finn background without Sami ethnicity) (6.9%), Kven, Finns and multi-ethnic Kven/Finns (without Norwegian ethnicity) (0.17%), and lastly the youth with ethnicity other than Sami, Norwegian or Kven/Finn (2.3%). There were no differences in socio-economic status between the Sami and non-Sami (χ^2 ^(1) = 0.00, p = .991), in both ethnic groups 56.2% were in the high socio-economic category.

The three different ethnic contexts differed in ethnic density of Sami youth. In the low density context (N = 2079), the percentage of Sami youth was 5.1; in the medium density context (N = 1156), there were 16.9% Sami; and in the high density context (N = 86), the percentage of Sami youth was 86. Only a quarter of all Sami youth had learned Sami language at home, while 72.2% identified themselves as Sami. Among the Sami, 76.9% (N = 346) lived in a rural area, while 72.3% (N = 2892) of the non-Sami youth lived in a rural area (χ^2 ^(1) = 4.266, p = .039).

### Ethnic differences in frequency of health service use

Sami and non-Sami youth used all health services with equal frequency (Table 1). There were no statistically significant interaction effects between gender and ethnicity concerning the school health service, general practitioner or the psychologist/psychiatrist. Table 1 shows the ethnic differences in health service use by the three different ethnic contexts. In the low density context, there were no differences between non-Sami and Sami in frequencies of use of the different health services. In the medium density context, Sami youth used a general practitioner more often than the non-Sami.

**Table 1:** Health service use during the past 12 months among non-Sami youth and Sami youth - overall frequencies and variations by different ethnic contexts

	**School Health Service**		**General Practitioner**		**Psychologist/Psychiatrist**	
			
	**Sami** **% (N)**	**Non-Sami** **% (N)**	**Sami** **% (N)**	**Non-Sami** **% (N)**	**Sami** **% (N)**	**Non-Sami** **% (N)**
Total	25.8 (114)	23.9 (944)	53.1 (234)	48.9 (1938)	5.9 (26)	5.6 (221)
Low density	24.8 (26)	23.7 (465)	47.6 (50)	50.7 (992)	5.6 (115)^1^	
Medium density	27.7 (53)	21.9 (208)	56.0 (107)*	47.9 (456)	5.8 (11)	5.7 (54)
High density	15.1 (13)^1^		53.6 (45)^1^		^2^	

Note: **p < 0.001 *p < 0.05; ^1 ^Total sum of both ethnic groups' use of health service; ^2 ^Not reported due to small N

Adolescents in urban areas used the school health service more than adolescents in rural areas (χ^2 ^(1) = 4.458, p = .035), while no differences were found between urban and rural areas in the use of the general practitioner and the psychologist/psychiatrist. There were no statistically significant interaction effects between urban/rural areas and ethnicity concerning the school health service, general practitioner or the psychologist/psychiatrist.

### Health service use, ethno-cultural factors and emotional/behavioural problems

#### School health service

Female gender and Sami self-identification were associated with an increase in using the school health service (Table 2). When emotional problems and behavioural problems were entered, youth with higher levels of hyperactivity problems and alcohol use had a higher probability of using the school health services than youth with lower problem levels. One of the interaction terms in step three was associated with visits to the school health service. Sami language competence influenced the use of the school health service dependent on the level of perceived discrimination. The likelihood of using the school health service was lower for speakers of Sami language with increasing levels of reported discrimination, compared with non-speakers with the same level of perceived discrimination.

**Table 2:** Stepwise logistic regression models of variables predicting the likelihood of seeking help from school health service, general practitioner and psychologist/psychiatrist

**Step and Variable**		**School Health**		**General Practitioner**		**Psychologist/Psychiatr**.	
				
		**OR**	**95%CI**	**OR**	**95%CI**	**OR**	**95%CI**
Step 1							
Gender:	Boys	1.00	Reference	1.00	Reference	1.00	Reference
	Girls	3.42**	2.72-4.30	1.64**	1.37-1.97	1.45	0.92-2.27
SES:	Low	1.00	Reference	1.00	Reference	1.00	Reference
	High	1.15	0.95-1.40	1.27*	1.08-1.49	0.77	0.53-1.12
Ethnicity:	Non-Sami	1.00	Reference	1.00	Reference	1.00	Reference
	Sami	0.56	0.29-1.08	1.45	0.88-2.40	0.54	0.13-2.21
Language:	Yes	1.00	Reference	1.00	Reference	1.00	Reference
	No	0.79	0.19-3.16	1.01	0.30-3.41	1.51	0.29-7.71
Sami self-id:	No	1.00	Reference	1.00	Reference	1.00	Reference
	Yes	2.78*	1.31-5.87	0.93	0.51-1.69	2.20	0.54-0.98
Perceived discrimination		1.10	0.93-1.29	0.92	0.80-1.05	1.05	0.79-1.40
Step 2							
Emotional problems		1.04	0.99-1.09	1.10**	1.05-1.15	1.29**	1.19-1.41
Conduct problems		1.00	0.94-1.08	0.97	0.91-1.03	1.11	0.98-1.26
Peer problems		0.95	0.89-1.02	0.96	0.91-1.02	1.15*	1.03-1.28
Hyperactivity problems		1.05*	1.00-1.11	1.04*	1.00-1.09	1.07	0.97-1.19
Alcohol use		1.21*	1.06-1.39	1.02	0.90-1.15	1.47*	1.14-1.90
Step 3							
Self-id. × Sami language		0.95	0.89-1.03	1.02	0.96-1.08	^1^	
Self-id × Discrimination		0.90	0.80-1.01	0.99	0.90-1.10	^1^	
Sami lang. × Discrimin.		1.13*	1.01-1.26	0.96	0.87-1.06	^1^	
Ethnicity × Emo. probl.		0.96	0.86-1.06	1.04	0.95-1.15	0.88	0.74-1.05
Ethnicity × Cond. probl		0.96	0.86-1.06	0.93	0.85-1.02	0.82*	0.67-0.99
Ethnicity × Hyperactivity		1.05	0.94-1.17	1.02	0.93-1.12	1.23	0.99-1.54
Ethnicity × Peer probl.		1.01	0.91-1.11	0.99	0.91-1.08	1.13	0.97-1.33
Ethnicity × Alcohol use		1.06	0.96-1.17	1.08	0.99-1.18	1.08	0.91-1.30

Note: **p < 0.001 *p < 0.05; Nagelkerke R^2 ^for school health service = 0.124; Nagelkerke R^2 ^for GP = 0.064; Nagelkerke R^2 ^for psychologist/psychiatrist = 0.165; ^1 ^variables not included in the model

#### General practitioner

Female gender and high socio-economic status were significantly associated with using the general practitioner (Table 2). When entering problem behaviours into the model, emotional problems and hyperactivity problems were positively associated with use of the general practitioner. None of the interaction variables were associated with visits to the general practitioner.

#### Psychologist/psychiatrist

None of the variables in the first step were significantly associated with use of the psychologist/psychiatrist (Table 2). In step two, the main effects of problems were explored, and the results showed that youth with emotional problems, peer problems and alcohol use were more likely to report having visited the psychologist/psychiatrist. In step three, the interaction between ethnicity and conduct problems was statistically significant. Non-Sami youth with conduct problems had a higher probability of using a psychologist/psychiatrist than Sami youth with conduct problems.

## Discussion

### Ethnic differences in health service use

There were no ethnic differences in overall frequency of health service use between Sami and non-Sami youth. However, in the medium density context, Sami youth used the general practitioner more often compared with the non-Sami. The similarities between the ethnic groups could be interpreted in several ways. Firstly, the finding could indicate that financial barriers and access of health services are similar among the ethnic groups in North Norway. Secondly, the finding could suggest that there is not more "health need" in the ethnic minority group. With a simple correspondence between actual use and ill-health, this would be one plausible interpretation. However, this relationship is more complicated; there are more factors than illness that influence whether health services are being used or not, for instance barriers specific to ethnic minorities [10]. Thirdly, the ethnic similarities in the pattern of health service use may suggest that the Sami minority youth have multicultural competence. Although the Norwegian nurse or doctor is culturally different in many aspects, the young person has the skills to manage this meeting without emotional discomfort or communication barriers. The informants in the present study were young and most of them had grown up in a multicultural setting, which may have contributed to the fact that they seemed to have good adaptation skills [33,34]. In sum, the results showed that the North Norwegian adolescents had good coping skills regarding help-seeking: equally often, Sami and non-Sami youth sought help when they experienced health problems. In addition, Sami youth seemed capable of coping with and managing cultural differences in their help-seeking process.

When the different ethnic contexts were explored separately, we found that Sami in the medium density context used the general practitioner more frequently than the non-Sami. Again, this could indicate more ill-health or psychosocial stress related to life in a marginalized Sami area. Exploring intra-group differences in behavioural problems in Sami youth, Kvernmo found that that Sami youth living in low density contexts or Norwegian-dominated areas reported more problems compared with Sami youth from Sami-dominated (high density) areas [5,8]. Sami youth from Norwegian-dominated contexts have experienced more deculturation, prejudice and discrimination compared with Sami youth from the Sami-dominated contexts. Kvernmo and Heyerdahl argued that the difference in mental health need stems from these factors [5]. The contextual difference found in the present study may reflect the need for health services being greater in the more assimilated Sami regions.

### Ethno-cultural factors and health service use

All the different ethno-cultural factors explored in this study did affect some aspects of health service use. Having a Sami background influenced the probability of using the mental health service for conduct problems; subjective Sami identity predicted school health service use; and lastly, Sami language competence acted as a barrier against use of the school health service if the degree of perceived discrimination was high.

The relationship between Sami self-identification and the school health service was found to be robust; it remained significant even after controlling for gender, socio-economic status and emotional/behavioural problems. By expressing Sami affiliation in daily life, one may expose oneself to more discrimination which can lead to psychosocial stress and the feeling of alienation. This finding may partly reflect health consequences of the negative aspects of being a member of a minority and having to face both structural and personal discrimination [8,35].

Speaking Sami language and experiencing ethnic discrimination together acted as a barrier against school health service use for Sami adolescents. Perhaps this finding indicates that some Sami youth do not wish to visit the school nurse due to lack of trust in the service or a lack of belief that it could be helpful for them [9,16]. Additionally, for the minority patient, fear of stigma and discrimination may be barriers against using health services [11,12].

Sami youth with conduct problems had a lower probability than the non-Sami of using a psychologist/psychiatrist. This specific finding may be an example of cultural influence on problem recognition. Social norms within the culture may encourage help-seeking for emotional problems, but not for behavioural problems. A recent study on parental reports on child problem behaviour comparing Sami and Norwegian parents, did in fact find that there were cultural differences in parental tolerance of problem behaviours. Sami parents had higher thresholds for identifying child behaviour as problematic, compared with the Norwegian parents [36].

### Behavioural/emotional problems, demographic factors and health service use

Emotional and behavioural distress was positively associated with health service use in our sample; this applied to both the primary health services and the specialist service for mental health. It seems that awareness of having problems increases the probability of seeking help from the health services [20,22]. There were some differences between the different help sources. The school health service was more strongly associated with hyperactivity problems and alcohol use than internalization problems and social problems. Problem behaviours most strongly associated with use of the general practitioner were emotional problems and hyperactivity problems. Emotional problems, social problems and alcohol use predicted use of the mental health services most strongly. These differences may be seen as adolescents having certain ideas about the specific suitability of the different professional help sources. Or, different service providers, parents or other adults may notice and act on some problem behaviours of youth more than others, and on behalf of the adolescents, they choose different services for different problem behaviours. Anyhow, professionals should be aware that for internalizing problems, the probability of seeking help from the school health service may be lower than for externalizing problems.

Girls used most of the health services significantly more than boys. This confirms a trend found in other youth health surveys [21,37,38]. In our sample, girls used the primary health services more than boys, but there were no gender differences in use of specialist health services for emotional or behavioural problems. The gender difference disappeared in the specialist health service, possibly due to the general practitioner acting as a "gatekeeper" to the specialist health service [20].

An unexpected finding was that use of the general practitioner varied by socio-economic status, even after controlling for behavioural and emotional problems. Adolescents having parents with high socio-economic status had a higher probability of seeking help from the general practitioner compared with adolescents from families of low socio-economic status. Previous research from Nordic countries found that the use of a general practitioner did not depend on parental education, while the use of specialist health services was more common in families with high education in parents [39-41]. Even though there are few socio-economic inequalities in North Norway, it seems that socio-economic background is an important factor to take into account when trying to understand why some adolescents do seek help and others do not.

### Limitations and strengths

A rather important limitation of the present study is that the adolescents were not asked whether they saw the health professionals because of emotional or behavioural problems. We found that youth with higher levels of symptoms were more likely to use health services, but we are not sure whether they used the services because of their psychological difficulties, or if there were other reasons. This means that conclusions about the relationship between problem behaviour and health service use should be reached with caution. Research has shown, however, that the prevalence of psychological problems in youth attending primary health services may be as high as 25% [42].

The distribution of respondents using a psychologist/psychiatrist was skewed; only about 5% of the total population had used this health service which results in low statistical power and increases the probability of type II errors.

The subscales of the SDQ-scale did not have very strong internal consistency. There are some potential causes for this [43]. Firstly, the homogeneity of the scale items may be low. Secondly, the low number of items in a subscale makes it less likely to get high alpha values. The discriminative validity of the SDQ could also be questioned. Hill and Hughes found that the SDQ subscales did not appear to discriminate very well between the constructs of emotional symptoms, conduct problems, hyperactivity and peer problems [44].

We had no possibility of controlling for availability of health services, except that we explored differences in use between rural and urban areas. There may be differences in baseline access and quality of services between different schools, different municipalities and different counties. Even so, we assumed that these potential differences were stable across the ethnic groups.

The major strengths of this study were the population based design, the high response rate and the representability of adolescents aged 15-16 years, including the Sami. Our findings add to prior knowledge by supplying information about the effect of culturally relevant variables on the help-seeking behaviour of the North Norwegian multiethnic youth population. Another strength is that we looked at self-reported *behaviour *as opposed to ratings of, for example, attitudes or opinions; we had hard data on how many times during the past year the youth had actually visited the health services.

## Conclusion

The present study was the first population based study on factors associated with health service use in the youth population of North Norway. It was also the first study to compare Sami and non-Sami youth's health service utilization. The main findings were that there were no overall ethnic differences in frequency of health service use. However, several ethno-cultural factors were found to influence health service use. Sami self-identification was strongly associated with school health service use and Sami youth living in a medium density context were more likely than non-Sami to use the general practitioner. Some possible barriers were also identified. Sami youth with conduct problems were less likely to be users of the psychologist/psychiatrist compared with their non-Sami peers. Youth who were Sami speakers and reported high perceived discrimination had a low probability of using the school health service. Finally, the present study demonstrated a relationship between health service use and self-reported emotional and behavioural difficulties in the multi-ethnic population of North Norwegian adolescents.

For ethnic minority youth, there may be culture-specific factors influencing help-seeking process; both barriers and factors increasing help-seeking may exist. The knowledge in this area is scarce. There is a great need for theoretical development in this area, as well as empirical studies identifying culture-specific factors influencing health service use. Factors affecting health service use should be considered when interventions are planned.

## Competing interests

The authors declare that they have no competing interests.

## Authors' contributions

ALT and MB participated in the literature review, in developing and generating hypotheses and statistical analyses, and drafted the manuscript. SK has been the project manager of The Norwegian Arctic Adolescent Health Study and responsible for the content of the questionnaire and the data collection, and has together with IS participated in the development of hypotheses, the statistical analyses and supervised the drafting of the manuscript.

All authors have read and approved the final manuscript.

## Pre-publication history

The pre-publication history for this paper can be accessed here:


